# Epigenetics of single-site and multi-site atherosclerosis in African Americans from the Genetic Epidemiology Network of Arteriopathy (GENOA)

**DOI:** 10.1186/s13148-022-01229-3

**Published:** 2022-01-17

**Authors:** Farah Ammous, Wei Zhao, Lisha Lin, Scott M. Ratliff, Thomas H. Mosley, Lawrence F. Bielak, Xiang Zhou, Patricia A. Peyser, Sharon L. R. Kardia, Jennifer A. Smith

**Affiliations:** 1 Department of Epidemiology, School of Public Health, University of Michigan, Ann Arbor, MI USA; 2 Memory Impairment and Neurodegenerative Dementia (MIND) Center, University of Mississippi Medical Center, Jackson, MS USA; 3 Department of Biostatistics, School of Public Health, University of Michigan, Ann Arbor, MI USA; 4 Survey Research Center, Institute for Social Research, University of Michigan, Ann Arbor, MI USA

**Keywords:** Methylation risk score, DNA methylation, Epigenetic age, Atherosclerosis, Calcification

## Abstract

**Background:**

DNA methylation, an epigenetic mechanism modulated by lifestyle and environmental factors, may be an important biomarker of complex diseases including cardiovascular diseases (CVD) and subclinical atherosclerosis.

**Methods:**

DNA methylation in peripheral blood samples from 391 African-Americans from the Genetic Epidemiology Network of Arteriopathy (GENOA) was assessed at baseline, and atherosclerosis was assessed 5 and 12 years later. Using linear mixed models, we examined the association between previously identified CpGs for coronary artery calcification (CAC) and carotid plaque, both individually and aggregated into methylation risk scores (MRS_CAC_ and MRS_carotid_), and four measures of atherosclerosis (CAC, abdominal aorta calcification (AAC), ankle–brachial index (ABI), and multi-site atherosclerosis based on gender-specific quartiles of the single-site measures). We also examined the association between four epigenetic age acceleration measures (IEAA, EEAA, PhenoAge acceleration, and GrimAge acceleration) and the four atherosclerosis measures. Finally, we characterized the temporal stability of the epigenetic measures using repeated DNA methylation measured 5 years after baseline (*N* = 193).

**Results:**

After adjusting for CVD risk factors, four CpGs (cg05575921(*AHRR*), cg09935388 (*GFI1*), cg21161138 (*AHRR*), and cg18168448 (*LRRC52*)) were associated with multi-site atherosclerosis (FDR < 0.1). cg05575921 was also associated with AAC and cg09935388 with ABI. MRS_CAC_ was associated with ABI (Beta = 0.016, *P* = 0.006), and MRS_carotid_ was associated with both AAC (Beta = 0.605, equivalent to approximately 1.8-fold increase in the Agatston score of AAC, *P* = 0.004) and multi-site atherosclerosis (Beta = 0.691, *P* = 0.002). A 5-year increase in GrimAge acceleration (~ 1 SD) was associated with a 1.6-fold (*P* = 0.012) increase in the Agatston score of AAC and 0.7 units (*P* = 0.0003) increase in multi-site atherosclerosis, all after adjusting for CVD risk factors. All epigenetic measures were relatively stable over 5 years, with the highest intraclass correlation coefficients observed for MRS_carotid_ and GrimAge acceleration (0.87 and 0.89, respectively).

**Conclusions:**

We found evidence of an association between DNA methylation and atherosclerosis at multiple vascular sites in a sample of African-Americans. Further evaluation of these potential biomarkers is warranted to deepen our understanding of the relationship between epigenetics and atherosclerosis.

**Supplementary Information:**

The online version contains supplementary material available at 10.1186/s13148-022-01229-3.

## Background

Cardiovascular diseases (CVD), including coronary heart disease, myocardial infarction, and peripheral artery disease, are the leading cause of death in the USA [1]. The CVD mortality burden is 21% higher in African-Americans compared to Whites despite modest decreases in racial disparities at the national level since 2005 [2]. Genetic factors, along with non-genetic risk factors, such as age, smoking, and hypertension, contribute to CVD. However, much of the variability in CVD, as well as the persistent causes of CVD disparities, remain unexplained.

Atherosclerosis, a chronic inflammatory age-related condition that develops over several decades, is a precursor for CVD and can occur in either the intimal or medial layers of the arterial wall [3–6]. The intima is the innermost layer consisting of a smooth endothelium layer covered by elastic tissue. The accumulation of lipids and fibrous material in the intima can occur at the coronary arteries and large arteries including the aorta [6]. Coronary artery calcification (CAC) is a strong predictor of incident CVD and coronary heart disease beyond traditional CVD risk factors [7–10]. Medial calcification occurs in the tunica media which consists of smooth muscles cells and elastic fibers. It affects lower limb arteries, in addition to the aorta, and is typically associated with peripheral artery disease [6]. Medial lesions are thought to calcify earlier than intimal ones and result in vascular stiffness and reduced vessel compliance [11, 12], and they may be also associated with CVD [8, 13]. Medial calcification increases with aging and is prevalent in individuals with chronic kidney disease and diabetes mellitus. The ankle–brachial pressure index ratio (ABI) can be used to assess medial calcification in the peripheral arteries [11].

Epigenetic mechanisms, including DNA methylation (DNAm), capture both genetic influences as well as the imprints of lifestyle and environmental exposures throughout the life course, and may have potential as biomarkers of CVD risk. Epigenetic age acceleration measures are DNA methylation-based markers of biological aging that are associated with late-life onset diseases and mortality [14–18]. Epigenetic age acceleration measures include first generation measures (HorvathAge [14] and HannumAge [15]), trained on chronological age, and more recent measures trained on biological and physiological markers and chronological age, such as PhenoAge [19] and GrimAge [17]. Previous studies of genome-wide DNA methylation profiles or epigenetic age acceleration measures have reported statistically significant associations between epigenetic markers and CVD [20–26]; however, a majority of these studies were in cohorts of European ancestry and/or were cross-sectional rather than longitudinal.

Only a few epidemiological studies have examined the association between DNAm and subclinical CVD or atherosclerosis [27, 28]. A recent cross-sectional transcriptome and epigenome analysis of atherosclerosis in 1208 participants from the Multi-Ethnic Study of Atherosclerosis (MESA) identified 82 differentially methylated CpGs associated with either CAC or carotid plaque score at false discovery rate (FDR) ≤ 0.1 [27]. The sample was comprised of 45.9% Caucasians, 21.5% African-Americans, and 32.6% Hispanics. Race-specific analyses showed that the directions of the methylation changes were generally consistent across these groups, although some sites were not significant for African-Americans and Hispanics [27]. The most significant CpG associated with carotid plaque, cg05575921, is located in the *AHRR* gene body and is a well-documented smoking marker [29–32].

In the current study, we evaluated the association between potential epigenetic markers of atherosclerosis and single- or multi-site atherosclerosis, assessed 5 and 12 years later, in 391 African-Americans from the Genetic Epidemiology Network of Arteriopathy (GENOA). Our atherosclerosis measures included CAC, abdominal aorta calcification (AAC), and ABI. Epigenetic markers included the previously identified CpGs for atherosclerosis [27], methylation risk scores (MRSs) derived from these CpGs, and four epigenetic age acceleration measures (Horvath (IEAA) [14], Hannum (EEAA) [15], PhenoAge (PhenoAA) [19], and GrimAge (GrimAA) [17]). Finally, we characterized the temporal stability of the epigenetic age acceleration measures and the MRSs using longitudinal measures of DNA methylation for a subset of the sample (*N* = 129).

## Methods

### Study sample

GENOA is a community-based study in Rochester, MN and Jackson, MS that was established to identify genes influencing blood pressure [33]. In the first phase of GENOA (Phase I: 1996–2001), sibships with at least two adults with clinically diagnosed essential hypertension before age 60 were recruited, and all siblings in the sibship were invited to participate regardless of hypertension status. Exclusion criteria included secondary hypertension, alcoholism or drug abuse, pregnancy, insulin-dependent diabetes mellitus, or active malignancy.

At baseline (Phase I), a total of 1583 non-Hispanic Whites (Rochester, MN) and 1854 African-Americans (Jackson, MS) were enrolled. In the second phase (Phase II: 2001–2005), all participants were invited for a second examination. Eighty percent of African-Americans (*N* = 1482) and 75% of non-Hispanic Whites (*N* = 1213) from Phase I returned. At Phase III (2009–2011), 752 African-Americans returned for a third examination. Demographic information, medical history, clinical characteristics, lifestyle factors, and blood samples were collected in each phase. This study includes 391 African-American participants from 277 sibships who had their DNA methylation measured in whole blood samples collected at Phase I and were seen in Phase III. For a subset of 129 participants that were seen in Phase III, DNA methylation was measured at both Phase I and Phase II. Written informed consent was obtained from all participants and approval was granted by participating institutional review boards (University of Michigan, University of Mississippi Medical Center, and Mayo Clinic).

### Study measurements

Height was measured by stadiometer and weight by electronic balance. Body mass index (BMI) was calculated as weight in kilograms divided by the square of height in meters. Smoking was categorized as current, former, or never. Resting systolic (SBP) and diastolic blood pressure (DBP) were measured by a random zero sphygmomanometer and a cuff appropriate for arm size. The second and third of three readings, after the participant sat for at least five minutes, were averaged for analysis [34]. Hypertension was defined having an average SBP ≥ 140 mmHg or DBP ≥ 90 mmHg, or current anti-hypertensive medication use. Type 2 diabetes status (T2D) was defined as having fasting blood glucose levels ≥ 126 mg/dL or self-reported physician-diagnosed diabetes and current diabetes medications use. Serum total cholesterol (TC), high-density lipoprotein cholesterol (HDL-C), and triglycerides (TGs) were measured by standard enzymatic methods on a Hitachi 911 Chemistry Analyzer (Roche Diagnostics, Indianapolis, IN). TC was adjusted for statin use as TC/0.8. Low-density lipoprotein cholesterol (LDL-C) was calculated using the Friedewald formula for individuals with TGs below 400 mg/dl [35].

### DNA methylation, epigenetic age acceleration, and methylation risk scores

Genomic DNA from 1106 African-American participants from Phase I and 304 from Phase II was extracted from stored peripheral blood leukocytes using AutoGen FlexStar (AutoGen, Holliston, MA) and DNA methylation was measured using the Infinium MethylationEPIC BeadChip. DNA methylation processing procedures have been previously described [36]. Briefly, sex mismatches and outliers were excluded using the shinyMethyl R package [37]. Probes with detection *P* value < 10^–16^ were considered to be successfully detected [38]. Samples and probes that failed a detection rate of at least 10% were removed. The Noob method was used for individual background and dye-bias normalization [39]. Regression on Correlated Probes method was used to adjust for the probe-type bias in the data [40]. White blood cell type proportions within the blood sample were estimated using Houseman’s method [41]. A total of 1100 samples from Phase I and 294 from Phase II were available after quality control.

We derived two MRSs based on the regression coefficients of CpG sites associated with CAC and/or carotid plaque at FDR ≤ 0.1 in MESA [27]. For CAC, 1 of the 16 CpGs reported in MESA (cg00889709) was not available in GENOA. For carotid plaque, 6 of the 68 CpGs reported in MESA (cg06126421, cg05951221, cg09768249, cg08170227, cg00913954, and cg08882503) were not available in GENOA. Methylation M values were first adjusted for batch effects (modeled as random effects of plate, row, and column) and white blood cell counts (modeled as fixed effects) by adding the residuals from the linear mixed model to the mean DNA methylation of each CpG. These values were then weighted by the previously reported effect sizes [27] and summed over the CpGs to calculate two MRSs, MRS_CAC_ based on 15 CpGs and MRS_carotid_ based on 62 CpGs. Two CpGs (cg07033253 and cg23661483) were associated with both CAC and carotid plaque in MESA and are included in both MRSs. Higher values of MRS correspond to greater risk of atherosclerosis.

Of the 75 total CpGs used to construct the two MRSs, approximately 13% are in CpG island regions, 33% in shore/shelf regions, 11% in promoter regions, 21% in enhancer regions, 50% in transcription factor binding sites, and 52% in DNase hypersensitivity sites. Detailed annotation of the individual CpGs has been previously described [27]. To investigate whether single-nucleotide polymorphisms (SNPs) proximal to the CpG sites may have substantially influenced methylation levels, we examined whether any of the CpGs overlapped with SNPs using the DMRcate package [42]. Out of the 75 CpGs, only 4 CpGs (cg02475408, cg13913475, cg15501219, and cg23848152) had SNPs with a minor allele frequency of at least 1% within 10 base pairs of the CpG site itself.

For the epigenetic age acceleration estimation, methylation beta values for Phase I and Phase II DNA methylation were uploaded to the online Horvath epigenetic age calculator to calculate DNAm age [43]. Four measures of epigenetic age—HorvathAge, HannumAge, PhenoAge, and GrimAge—were estimated. Intrinsic epigenetic age acceleration (IEAA) is based on the regression residuals from a model of chronological age and blood cell counts and HorvathAge as the outcome [14, 44]. Extrinsic epigenetic age acceleration (EEAA) is derived similarly based on the HannumAge but incorporates weighted averages of three white blood cell types (naïve cytotoxic T cells, exhausted cytotoxic T cells, and plasmablasts) [15, 44]. PhenoAA is a residual measure from a model of phenotypic age, calculated based on clinical measures such as albumin, creatinine, and white blood cell counts, as well as chronological age [19]. GrimAA is based on the residuals from a model of GrimAge and chronological age. GrimAge is a composite biomarker of smoking pack-years and seven surrogate measures of plasma proteins selected for their significant association with time-to-death. The components are adrenomedullin (ADM), beta-2-microglobulin, cystatin C, GDF-15, leptin, plasminogen activator inhibitor antigen type 1 (PAI-1), and tissue inhibitor metalloproteinases 1 (TIMP-1) [17].

### Gene expression measures

Gene expression levels in GENOA African-American participants (N = 1233) were measured in Epstein–Barr virus (EBV) transformed B-lymphoblastoid cell lines using the Affymetrix Human Transcriptome Array 2.0. Quality control was done using the Affymetrix Expression Console provided by Affymetrix. All array images passed visual inspection and the Affymetrix CEL files were normalized using the Robust Multichip Average algorithm in the Affymetrix Power Tool software [45]. Adjustment for batch effects and other technical covariates was done using Combat [46] and mapping of probes to genes was done using the Brainarray custom CDF version 19 [47]. After quality control, a total of 17,616 autosomal protein coding genes for 1205 participants were available for analysis.

### Atherosclerosis measurements

Computed tomography (CT) imaging conducted at Phase III was used to quantify calcification in the coronary arteries and the abdominal aorta. CT images were read by trained technologists and the amount of calcified plaque was calculated by multiplying each lesion area weighted by attenuation based on the maximum Hounsfield units in the lesion on a TeraRecon Aquarius Workstation (TeraRecon, San Mateo, CA). The amount of calcification was quantified using the Agatston score [48]. Both CAC and abdominal aorta calcification (AAC) were natural log-transformed as ln (score + 1) when examined individually as outcomes.

ABI was used to quantify atherosclerosis in the peripheral arteries. Details about the ABI measurement conducted at Phase II have been previously described [49]. Briefly, a Doppler ultrasonic instrument (Medisonics, Minneapolis, MN) was used to detect the pulse at each arm and ankle using appropriately sized blood pressure cuffs. ABI was calculated as the systolic blood pressure at each ankle site divided by the higher of the two brachial pressures. The lower of the average ABIs from both legs was used. Individuals with ABI > 1.50 were excluded as they may have non-compressible arteries.

Multi-site atherosclerosis score was defined similarly to that described by Zhao et al. [50]. Both CAC and AAC were scored separately as follows: 0 if not detectable, or for those with detectable calcification as a score between 1 and 4 according to gender-specific quartiles of each measure. ABI was scored between 0 and 4 for the highest to lowest gender-specific quartiles for ABI < 1.0, 0 for 1 ≤ ABI < 1.4, and 1 if ABI ≥ 1.4 and ≤ 1.50. The multi-site atherosclerosis score was then calculated as the sum of the three measures (range 0–12) and was modeled as a continuous outcome.

### Statistical analysis

Outliers beyond 5 standard deviations from the mean of the outcome and epigenetic measures were removed. We calculated the Pearson correlation coefficients between the single-site atherosclerosis measures, and among the epigenetic age acceleration measures and MRSs. We also calculated the correlations between the individual GrimAge components and the MRSs.

We used linear mixed models that account for familial relatedness to assess the association between the previously identified atherosclerosis-associated CpGs in MESA and MRSs derived from these CpGs (predictors) and the single- or multi-site atherosclerosis measures (outcomes). The minimally adjusted model (Model 1) was adjusted for age at baseline, sex, first 4 genetic principal components (PCs), and time between the measures. The time covariate was calculated based on the age difference of the participants at the time of atherosclerosis assessment (Phase II or Phase III) and DNA methylation assessment (Phase I). For models of multi-site atherosclerosis, we included two time covariates—the age difference between Phase III and Phase I, and an additional covariate for the age difference between Phase III and Phase II—to account for the differences in the assessment times of ABI (at Phase II) and CAC and AAC (at Phase III). Model 2 was additionally adjusted for smoking status at baseline, and Model 3 was further adjusted for baseline CVD risk factors (T2D status, hypertension status, BMI, and statin-adjusted total cholesterol levels).

For CpGs associated with at least one of the atherosclerosis measures in Model 1 at FDR < 0.1, we investigated for association with cis-gene expression (± 1 Mb for each CpG). For that, we fit a linear mixed model adjusted for age, sex, time between measures, and the first 4 genetic PCs. For genes significantly associated with DNA methylation at FDR < 0.1, we investigated their association with the atherosclerosis measures using a linear mixed model approach with the same covariates.

We used a similar procedure as described above to assess the association between the four epigenetic age acceleration measures and atherosclerosis, except that genetic PCs were not included as covariates. This approach of not adjusting for genetic PCs is consistent with other studies and allows for comparability of findings. As sensitivity analysis, we also performed this analysis after adjusting for the first four genetic PCs. We additionally carried out sensitivity analyses adjusting for white blood cell counts for significant associations with PhenoAA and GrimAA to assess confounding by changes in blood cell composition [17, 19, 51]. For GrimAA, we investigated the association between the individual GrimAge components and atherosclerosis to identify components that may be driving the association between GrimAA and atherosclerosis or that outperform the overall GrimAA measure itself. For this analysis, GrimAge components were scaled and centered. Models were adjusted for age, sex, time between measures, and white blood cell counts.

For the subset of individuals with repeated DNA methylation measurements, we used chi-square and t tests as appropriate to compare the demographic and clinical characteristics of the subset to individuals without the repeated measurements. We used linear mixed-effect models adjusted for age and sex to calculate the intraclass correlation coefficients (ICC) between Phases I and II for the methylation risk scores and the epigenetic age acceleration measures. Parametric bootstrapping (1000 iterations) was used to calculate the 95% confidence intervals of the ICC coefficients [52, 53]. Additionally, for these participants, we assessed the associations between all of the epigenetic biomarkers at Phase II and atherosclerosis after adjusting for age, sex, time between measures (when applicable), first 4 genetic principal components, smoking status, hypertension status, diabetes status, body mass index, and total cholesterol levels adjusted for statin use.

Statistical tests were two-sided. False discovery rate (FDR) [54] of 0.1 was considered significant for the associations between the individual CpGs and the atherosclerosis measures. A Bonferroni adjusted *P* value of < 0.025 (for MRS association analyses) and *P* < 0.0125 (for epigenetic age acceleration association analyses) were considered significant. Analyses were conducted in R (Version 3.4.1) [55], using the lme4 [56], and rptR [53] packages.

## Results

### Sample characteristics

Baseline characteristics of the participants are shown in Table 1. The mean age of the participants was 56 years (SD = 9.0) at Phase I. Women comprised about 76% of the sample. Phase III was on average 12 years (SD = 1.2) after Phase I. About 64% of the participants had hypertension and 16% had T2D at Phase I. Distributions of the atherosclerosis measures and MRSs are shown in Additional file 1: Figs. S1 and S2, respectively. For MRS_CAC_, one outlier beyond 5 SD from the mean was removed from the analysis.

**Table 1:** Descriptive characteristics of GENOA African-Americans

Characteristics^a^	Overall (*N* = 391)
Females (%)	297 (76.0%)
Age at Phase I (years)	56.0 ± 9.0
Age at Phase III (years)	68.0 ± 8.4
*Cardiovascular risk factors at Phase I*	
Smoking status	
Never (%)	241 (61.6%)
Former (%)	93 (23.8%)
Current (%)	57 (14.6%)
Body mass index (kg/m^2^)	31.4 ± 6.2
Hypertension	249 (63.7%)
Type 2 diabetes	63 (16.1%)
Total cholesterol^b^ (mg/dl)	208.56 ± 50.4
Low-density lipoprotein cholesterol^c^ (mg/dl)	121.8 (42.8)
Statin use (%)	18 (4.6%)
*Atherosclerosis measures at Phase III*	
Coronary artery calcification score, median (IQR)	18.7 (0–195.7)
Abdominal aorta calcification score, median (IQR)	500.8 (18.9–1800.5)
Ankle–brachial index, median (range)^d^	0.985 (0.474–1.274)
Multi-site atherosclerosis score, median (IQR) (range 0–12)	5 (2–7)
*Methylation risk scores at Phase I*	
MRS_CAC_^e^	0.92 ± 0.85
MRS_carotid_	− 3.52 ± 0.81
*DNAm age measures at Phase I*	
HorvathAge (years)	52.8 ± 8.9
HannumAge (years)	46.2 ± 9.5
PhenoAge (years)	42.7 ± 11.5
GrimAge (years)	52.8 ± 7.9

*IQR* interquartile range, *DNAm* DNA methylation

^a^Means ± standard deviation

^b^Adjusted for statin use as total cholesterol/0.8

^c^Calculated using the Friedewald formula for participants with triglycerides < 400 mg/dl (*N* = 386)

^d^Measured at Phase II (mean: 5.2 ± 1.3 years from Phase I)

^e^*N* = 390, after removing one outlier beyond 5 standard deviation units from the mean

### Correlations among the atherosclerosis measures, epigenetic age acceleration measures, and methylation risk scores

CAC and AAC were correlated at *r* = 0.57 (*P* < 2.2 × 10^–16^). ABI was negatively correlated with AAC (*r* = − 0.14, *P* = 0.004), but only weakly correlated with CAC (*r* = − 0.08, *P* = 0.09). Additional file 1: Table S1 shows the correlation between the epigenetic predictors. The correlation between the acceleration measures was weak to moderate (*r* range 0.21–0.46). The two MRSs were correlated at *r* = 0.32 (*P* = 1.55 × 10^–10^). Most of the epigenetic age acceleration measures and MRSs were weakly correlated (*r* range 0.09–0.29), except for GrimAA and MRS_carotid_ which were correlated at *r* = 0.68 (*P* = 2.2 × 10^–16^). Scatterplots of the MRSs against the epigenetic age acceleration measures are shown in Additional file 1: Fig. S3. Of the GrimAge components, smoking pack-years was slightly more correlated with MRS_carotid_ than the overall GrimAA measure (*r* = 0.75, *P* = 2.2 × 10^–16^), with the second highest correlation between MRS_carotid_ and PAI-1 (*r* = 0.26, *P* = 2.9 × 10^–7^).

### Associations between previously identified atherosclerosis-associated CpGs, cis-gene expression, and atherosclerosis

CAC, AAC, ABI, and multi-site atherosclerosis were associated with 21, 15, 14, and 42 CpGs in Model 1, respectively (FDR < 0.1). The significant regression results are shown in Additional file 1: Table S2. The majority of these sites were carotid plaque-associated CpGs in MESA. Additional file 1: Table S3 shows the regression results for the CpGs that remained significantly associated with atherosclerosis after adjustment for CVD risk factors (Model 3) in GENOA. After adjusting for CVD risk factors, 4 CpGs (cg05575921 (*AHRR*), cg09935388 (*GFI1*), cg21161138 (*AHRR*), and cg18168448 (*LRRC52*)) remained significantly associated with multi-site atherosclerosis. CpG cg05575921 was also associated with AAC and cg09935388 with ABI (FDR < 0.1). All of these CpGs were carotid plaque-associated CpGs in MESA. Hypermethylation at all of the significant CpGs (FDR < 0.1 in Model 3) was associated with decreased multi-site atherosclerosis. Significant CpGs in Model 3 explained between 5.5% and 11.2% of the variability of multi-site atherosclerosis, cg05575921 explained 6.9% of the variability of AAC, and cg09935388 explained 11.1% of the variability in ABI.

To investigate any regulatory effects on gene expression by the significant CpGs, we examined the association between CpGs shown in Additional file 1: Table S1 and cis-gene expression (± 1 Mb). DNA methylation at cg18168448 was associated with decreased expression of *ALDH9A1* and increased expression of ENSG00000236364 at FDR < 0.1 (Additional file 1: Table S4). Increased *ALDH9A1* expression was associated with higher CAC (*P* = 0.005) and marginally higher multi-site atherosclerosis (*P* = 0.054). DNA methylation at cg03636183 was associated with decreased gene expression of *F2RL* which was associated with CAC (*P* = 0.017).

### Associations between methylation risk scores and atherosclerosis

Table 2 shows the associations between MRS_CAC_ and MRS_carotid_ and single- or multi-site atherosclerosis measures. The beta coefficients shown correspond to the change in the atherosclerosis measures associated with a 1-unit increase in MRS. In Model 1, MRS_CAC_ was associated with log-transformed AAC (Beta = 0.364, 95%CI 0.050–0.778, *P* = 0.023) but the association was not significant after adjusting for CVD risk factors (Models 2 and 3). MRS_CAC_ was associated with higher ABI, indicative of less peripheral artery disease, and the associations were significant after adjusting for CVD risk factors (Beta = 0.016, 95%CI 0.004–0.028, *P* = 0.006 in Model 3). The MRS_CAC_ explained 1.2%, 1.9%, 14.8%, and 0.8% of the variability of CAC, AAC, ABI, and multi-site atherosclerosis after adjusting for CVD risk factors.

**Table 2:** Association between methylation risk scores and atherosclerosis measures in GENOA African-Americans

Outcome	Methylation risk score	Model 1			Model 2			Model 3		
		Beta	SE	*P*	Beta	SE	*P*	Beta	SE	*P*
Coronary artery calcification score (CAC)^a^	MRS_CAC_	0.262	0.146	0.074	0.153	0.173	0.295	0.138	0.139	0.318
	MRS_carotid_	**0.700**	**0.155**	**8.66 × 10**^**–6**^	0.425	0.199	0.033	0.411	0.191	0.032
Abdominal aorta calcification score (AAC)^a^	MRS_CAC_	**0.364**	**0.160**	**0.023**	0.175	0.154	0.258	0.173	0.151	0.252
	MRS_carotid_	**1.046**	**0.167**	**1.10 × 10**^**–9**^	**0.580**	**0.211**	**0.006**	**0.605**	**0.208**	**0.004**
Ankle–brachial index (ABI)	MRS_CAC_	0.013	0.006	0.025	**0.015**	**0.006**	**0.008**	**0.016**	**0.006**	**0.006**
	MRS_carotid_	− **0.014**	**0.006**	**0.021**	− 0.009	0.008	0.275	− 0.007	0.008	0.400
Multi-site atherosclerosis score	MRS_CAC_	0.317	0.170	0.064	0.145	0.165	0.382	0.127	0.162	0.433
	MRS_carotid_	**1.165**	**0.177**	**1.65 × 10**^**–10**^	**0.718**	**0.225**	**0.002**	**0.691**	**0.223**	**0.002**

Model 1 is adjusted for age, sex, time between measures, and the first 4 genetic principal components

Model 2 is adjusted for Model 1 covariates and smoking status

Model 3 is adjusted for Model 2 covariates, hypertension status, diabetes status, body mass index, and total cholesterol levels adjusted for statin use

Beta is the change in the atherosclerosis measure associated with a 1 unit increase in the MRS

P values significant after Bonferroni correction (*P* < 0.025) are shown in bold font

^a^Coronary artery and abdominal aorta calcification scores were transformed as ln[(CAC + 1)] and ln[(AAC + 1)]

MRS_carotid_ was associated with all of the atherosclerosis measures in Model 1 (Table 2). Associations remained significant for AAC after adjusting for smoking (Model 2) and other traditional CVD risk factors (Model 3), where a one unit increase in MRS_carotid_ was associated 0.605 units increase in log-transformed AAC (95%CI 0.197–1.013). This is equivalent to an approximately 1.8-fold increase in the Agatston score of AAC. Similarly, the associations between MRS_carotid_ and multi-site atherosclerosis remained significant after adjusting for CVD risk factors. A one unit increase in MRS_carotid_ was associated with approximately 0.7 units (95%CI 0.25–1.13) increase in the multi-site atherosclerosis score after adjusting for CVD risk factors (Model 3). The MRS_carotid_ explained 7.8%, 5.3%, 2.1%, and 9.4% of the variability of CAC, AAC, ABI, and multi-site atherosclerosis after adjusting for CVD risk factors.

### Associations between epigenetic age acceleration and atherosclerosis

Associations between the epigenetic age acceleration measures and atherosclerosis are shown in Table 3. PhenoAA was associated with multi-site atherosclerosis in Model 1, but the association attenuated after adjusting for CVD risk factors. GrimAA was positively associated with all measures of atherosclerosis in the minimally adjusted model (Model 1) and remained significant after adjusting for CVD risk factors for AAC and multi-site atherosclerosis. A 5-year increase in GrimAA (~ 1 SD) was associated with a 1.6-fold (95%CI 1.11–2.25) increase in the Agatston score of AAC and 0.7 units (95%CI 0.33–1.07) increase in multi-site atherosclerosis score in Model 3. Increased GrimAA was nominally associated with lower ABI (higher atherosclerosis), but the association was not significant after accounting for multiple testing. The effect estimates were unchanged after adjusting for the first 4 PCs. Additional file 1: Table S5 shows the associations for PhenoAA and GrimAA after adjusting for white blood cell counts. The associations between GrimAA and AAC and multi-site atherosclerosis in Model 3 slightly attenuated after adjusting for white blood cells counts but remained significant at *P* < 0.05.

**Table 3:** Associations between epigenetic age acceleration and atherosclerosis measures in GENOA African-Americans

Outcome	Epigenetic age acceleration	Model 1			Model 2			Model 3		
		Beta	SE	*P*	Beta	SE	*P*	Beta	SE	*P*
Coronary artery calcification score (CAC)^a^	IEAA	0.016	0.027	0.555	0.008	0.026	0.750	− 0.008	0.025	0.741
	EEAA	0.018	0.023	0.436	0.012	0.022	0.589	0.003	0.021	0.901
	PhenoAA	0.034	0.018	0.056	0.023	0.017	0.191	0.010	0.017	0.532
	GrimAA	**0.149**	**0.028**	**2.04 × 10**^**–7**^	**0.105**	**0.034**	**0.002**	0.074	0.033	0.025
Abdominal aorta calcification score (AAC)^a^	IEAA	− 0.016	0.029	0.591	− 0.025	0.028	0.366	− 0.040	0.027	0.139
	EEAA	0.027	0.025	0.285	0.024	0.024	0.311	0.018	0.024	0.437
	PhenoAA	0.046	0.019	0.018	0.033	0.018	0.073	0.028	0.018	0.133
	GrimAA	**0.188**	**0.031**	**2.94 × 10**^**–9**^	**0.106**	**0.036**	**0.004**	**0.092**	**0.036**	**0.012**
Ankle–brachial index (ABI)	IEAA	− 0.001	0.001	0.562	0	0.001	0.644	− 0.001	0.001	0.580
	EEAA	0	0.001	0.649	0	0.001	0.755	0	0.001	0.795
	PhenoAA	− 0.001	0.001	0.076	− 0.001	0.001	0.133	− 0.001	0.001	0.149
	GrimAA	− **0.004**	**0.001**	**0.002**	− 0.003	0.001	0.024	− 0.003	0.001	0.026
Multi-site atherosclerosis score	IEAA	0.021	0.031	0.489	0.011	0.029	0.713	− 0.002	0.029	0.942
	EEAA	0.027	0.027	0.318	0.021	0.026	0.417	0.013	0.025	0.613
	PhenoAA	**0.067**	**0.020**	**0.001**	**0.052**	**0.020**	**0.008**	0.044	0.019	0.023
	GrimAA	**0.231**	**0.032**	**4.59 × 10**^**–12**^	**0.158**	**0.038**	**4.58 × 10**^**–5**^	**0.140**	**0.038**	**3.01 × 10**^**–4**^

*IEAA* intrinsic epigenetic age acceleration, *EEAA* extrinsic epigenetic age acceleration, *PhenoAA* PhenoAge acceleration, *GrimAA* GrimAge acceleration

Model 1 is adjusted for age, sex, and time between measures

Model 2 is adjusted for Model 1 covariates and smoking status

Model 3 is adjusted for Model 2 covariates, hypertension status, diabetes status, body mass index, and total cholesterol levels adjusted for statin use

Beta is the change in the atherosclerosis measure associated with a 1 unit increase in the epigenetic age acceleration measure

P values significant after Bonferroni correction (*P* < 0.0125) are shown in bold font

^a^Coronary artery and abdominal aorta calcification scores were transformed as ln[(CAC + 1)] and ln[(AAC + 1)]

The associations between the DNA methylation-based surrogate measures comprising GrimAge and atherosclerosis are shown in Additional file 1: Table S6. Associations were adjusted for age, sex, time between measures, and white blood cell counts. All components were associated with at least one single- or multi-site atherosclerosis measure, with the exception of leptin and TIMP-1 (*P* < 0.05). DNAm smoking pack-years was associated with each of the atherosclerosis measures and was the most significant predictor of CAC, AAC, and multi-site atherosclerosis, with consistent direction of effects across all of the measures. Compared to the corresponding model of GrimAA (Additional file 1: Table S3; Model 1), DNAm smoking pack-years was more significantly associated with single- and multi-site atherosclerosis, with greater magnitude of effects, than the overall GrimAA measure.

### Longitudinal correlation of methylation risk scores and epigenetic age acceleration measures between Phases I and II

Compared to the remaining sample, the 129 individuals with longitudinal measures of DNA methylation were younger (mean age of 53.4 vs. 57.3 years, *P* < 0.001), had a lower multi-site atherosclerosis score (mean of 4.51 vs. 5.26, *P* = 0.026) and a lower PhenoAA (mean of − 1.11 vs. 0.41, *P* = 0.043)*.* All the remaining epigenetic and atherosclerosis measures were similar across both samples (*P* > 0.05). The mean time difference between the DNA methylation measurements was 5.5 years (standard deviation = 1.1 years, range 2.5–7.4). All of the epigenetic measures were relatively stable between Phases I and II, with both MRS_carotid_ and GrimAA showing the highest stability (ICC > 0.8) (Table 4). MRS_carotid_ derived at Phase II was associated with consistent effect direction and larger effect magnitude for log-transformed AAC (Beta = 1.08, 95% CI 0.380–1.78, *P* = 0.003) and was marginally associated with multi-site atherosclerosis (Beta = 0.811, 95% CI 0.090–1.53, *P* = 0.030) compared to the full sample analysis using Phase I MRSs (Table 2, Model 3). Neither MRS was associated with CAC or ABI at Phase II (Additional file 1: Table S7). Additionally, none of the epigenetic age acceleration measures at Phase II were associated with atherosclerosis.

**Table 4:** Inter-individual correlations for the epigenetic measures (Phases I and II) in GENOA African-Americans (*N* = 129)

Measures	Intraclass correlation coefficient (95%CI)
MRS_CAC_	0.771 (0.689–0.833)
MRS_carotid_	0.867 (0.821–0.905)
IEAA	0.726 (0.635–0.802)
EEAA	0.799 (0.726–0.854)
PhenoAA	0.676 (0.569–0.758)
GrimAA	0.888 (0.848–0.921)

*IEAA* intrinsic epigenetic age acceleration, *EEAA* extrinsic epigenetic age acceleration, *PhenoAA* PhenoAge acceleration, *GrimAA* GrimAge acceleration

## Discussion

In this study of African-Americans with a high prevalence of hypertension, we examined the association between peripheral blood DNA methylation patterns, measured at baseline, and multi-site atherosclerosis assessed five and 12 years later. After adjusting for CVD risk factors, a total of 4 CpGs were associated with multi-site atherosclerosis, with one of them additionally associated with ABI (cg09935388) and another associated with AAC (cg05575921). DNA methylation at cg18168448 was associated with *cis*-gene expression changes that were also associated with CAC in GENOA. An aggregate risk score of CpGs, MRS_CAC_, was associated with ABI and MRS_carotid_ was associated with AAC and multi-site atherosclerosis after adjusting for CVD risk factors. GrimAA was the only epigenetic age acceleration measure associated with atherosclerosis. GrimAA and MRS_carotid_ were moderately correlated with each other, and both measures were relatively stable over 5 years in a small subset of participants with repeated DNA methylation measurement. Our findings show evidence of association between DNA methylation sites, previously identified in a cross-sectional analysis, and atherosclerosis assessed 5 and 12 years later. Additionally, our findings show evidence of overlap in the association between these CpGs and atherosclerosis at different vascular sites. We also report an association between age-related methylation changes, as measured by the epigenetic clocks, and vascular calcification.

After adjusting for CVD risk factors, four CpG was associated multi-site atherosclerosis at FDR < 0.1. The direction of effects between the individual CpG sites and atherosclerosis in our study were consistent with MESA, where hypomethylation was associated with increased carotid plaque [27]. All 4 CpGs are in gene body regions. Both cg05575921 and cg21161138 are at transcription factor binding sites. CpG cg05575921 maps to an enhancer chromatin state and cg21161138 to a weak repressed chromatin state in primary mononuclear cells from peripheral blood (E062) using chromHMM tracks from Roadmap Epigenomics [57, 58]. CpG cg09935388 maps to a CpG island, a DNase hypersensitive hotspot, and a weak transcribed chromatin state while cg18168448 maps to a repressed chromatin state [57, 58].

Three of the significant CpGs (cg05575921, cg09935388, and cg21161138) were previously found to be associated with smoking [29–32]. When we examined the methylation levels of these three CpGs by smoking status in GENOA, we observed a dose response association where current smokers had the lowest methylation levels and never smokers had the highest methylation levels (Additional file 1: Fig. S4). Both cg05575921 and cg21161138 are located in the *AHRR* gene body. *AHRR* is an aryl hydrocarbon receptor repressor, which among other roles, inhibits the metabolism of polycyclic aromatic hydrocarbons and dioxins by competing with AHR [59, 60]. The significant association between these CpGs and atherosclerosis after adjusting for smoking status in our study could potentially be related to residual confounding, errors in self-reporting of smoking, and/or inter-individual sensitivities to smoking with lasting biological effects. In a previous analysis in MESA that used a candidate gene approach to assess the association between CpGs in *AHRR* and atherosclerosis, hypomethylation at cg05575921 (*P* = 3.08 × 10^−10^) and cg21161138 (*P* = 7.73 × 10^−8^) was significantly associated with carotid plaque score [61]. Similar to our findings, the association with cg05575921 in MESA remained significant after adjusting for self-reported smoking exposure, urinary cotinine, and other CVD risk factors, and remained significant in stratified analysis of former smokers and current smokers but not never smokers. Other studies have also reported evidence of cg05575921 differential methylation by air pollution in adults [62], by maternal smoking in neonates [63, 64], and by smoking in atherosclerotic plaque specimens [65]. Similarly, cg09935388, located in the growth factor independent 1 transcriptional repressor gene (*GFI1*) gene body region, has been found to be associated with smoking [32, 66, 67] and exposure to maternal smoking in fetuses [68, 69]. *GFI1* encodes a nuclear zinc finger protein that plays a role in hematopoiesis, oncogenesis, and in controlling histone modification as part of a complex with other cofactors [70, 71].

DNA methylation at cg18168448 was also associated with significantly higher multi-site atherosclerosis (FDR = 0.063). The direction of association was consistent for the single-site atherosclerosis measures although it did not reach statistical significance. CpG cg18168448 is located in the first exon region of the leucine-rich repeat-containing protein 52 (*LRRC52*), which plays a role in the regulation of voltage-gated potassium channel activity. We found evidence of *cis*-gene expression changes at this CpG, cg18168448, in addition to cg03636183. Our findings in EBV transformed B-lymphoblastoid cell lines are complimentary to those reported previously in monocytes [27]. DNA methylation at cg18168448 was associated with decreased expression of the aldehyde dehydrogenase 9 family member A1 (*ALDH9A1*) and increased expression of ENSG00000236364, which is antisense to *UCK2*. *ALDH9A1* encoded enzyme has high activity for oxidation of gamma-aminobutyraldehyde and other amino aldehydes. *UCK2* encodes a pyrimidine ribonucleoside kinase, which catalyzes the phosphorylation of uridine and cytidine. DNA methylation at cg0363183 located in the body region of F2R like thrombin or trypsin receptor 3 (*F2RL3*) was associated with significant decreased gene expression of that gene. *F2RL3* plays a role in blood coagulation and inflammation, and methylation at cg0363183 has been previously found to be associated with smoking [72], cardiometabolic traits [73], coronary artery disease [74], and morality [75]. The genes corresponding to the 4 significant CpGs and those identified in the cis-gene expression analysis are expressed in low to medium levels in multiple tissue types, with the exception of *LRRC52* which is mainly expressed in the testis [76]. *AHRR*, *GF1*, *ALDH9A1*, ENSG00000236364, and *F2RL3* have at least a low level of expression in the arteries, heart, and whole blood.

In our study, MRS_CAC_ was associated with AAC and ABI and the association fully attenuated after adjusting for CVD risk factors for AAC. MRS_carotid_ was associated with all of the atherosclerosis measures in Model 1 and AAC and the derived multi-site atherosclerosis scores remained significant after adjusting for CVD risk factors. Two of the significant CpGs in MRS_carotid_ explained a slightly higher percent of the variability of multi-site atherosclerosis compared to that explained by MRS_carotid,_ potentially due to added noise generated by including CpGs not as strongly associated with atherosclerosis in African-Americans. It is not clear why carotid plaque-associated CpGs were better predictors of atherosclerosis than CAC-associated ones in our sample. One notable finding in MESA was that both the transcriptome signature of AT-rich interaction domain 5B (*ARID5B*) and a cg25953130 site in the gene were associated with CAC and carotid plaque atherosclerosis measures [27]. In GENOA, cg25953130 was only nominally associated with multi-site atherosclerosis after adjusting for CVD risk factors (Beta = − 0.596, 95%CI − 0.063 to − 1.13, *P* = 0.029), but the effect direction was consistent across studies. In MESA, out of seven CpGs associated with CAC (FDR < 0.05) in the full sample, only 3 were significant in the African-American sub-sample, although the magnitude and direction of effect were consistent across ancestry groups. Also, despite having similar age distributions, MESA multi-ethnic participants had a higher median CAC score than African-Americans in GENOA, and MESA had also a lower percentage of females. The prevalence of CAC in GENOA was low (median: 18.7 and IQR 0–195.7) with about 40% of the sample having an Agatston score of zero. In MESA, participants had a median CAC score of 46 (IQR 0–305), and the proportion with no detectable CAC was not reported. This is consistent with evidence from epidemiological studies showing that African-Americans tend to have lower calcification in the coronary arteries compared to Whites [77–79]. Hence, the CpGs included in the MRS_CAC_ could be related to a more extreme form of the trait versus the lower atherosclerosis burden seen in GENOA. Another difference between MESA and GENOA is that the methylation signature in MESA was measured in monocytes, which have a well-established role in atherogenesis [80, 81], while the DNA methylation in GENOA was measured in all white blood cells. This could have the effect of diluting the associations observed in MESA if some cell types have different methylation patterns compared to monocytes. Additionally, the ICC of MRS_CAC_ was low, which could indicate a lower stability of methylation at these sites. This could be particularly relevant to GENOA, as we assessed the association with CAC 12 years after DNA methylation measurement, while in MESA methylation and atherosclerosis were assessed concurrently.

GrimAA was the only epigenetic age acceleration measure associated with atherosclerosis in GENOA after adjusting for CVD risk factors. Increased GrimAA, indicative of increased biological aging, was associated with higher atherosclerosis. Very few other studies have examined epigenetic age associations with subclinical measures of CVD. In a cross-sectional analysis of 2500 African-Americans from the Atherosclerosis Risk in Communities (ARIC) study, a 5-year increase in both the Horvath and the Hannum acceleration measures was associated with an approximately 0.01 mm increase in carotid intima thickness [28]. We did not find associations between the Horvath and Hannum measures we evaluated (IEAA and EEAA) and atherosclerosis. Epigenetic aging measures include different CpG sites, differ in how they were trained, and are hypothesized to capture different biological processes and aspects of aging [16, 51]. Both GrimAA and PhenoAA were trained using longitudinal data, making them better predictors of aging-related outcomes [82, 83]. Two recent studies of participants of European ancestry have found that GrimAA outperforms the other acceleration measures in its association with incident CVD [25, 26] and all-cause mortality [25, 84]. Our reported associations attenuated slightly after adjusting for white blood cell counts but remained significant, suggesting that the effects were not mediated by blood cell composition. In our study, GrimAA was correlated with MRS_carotid_, with the smoking pack-years component of GrimAge being slightly more correlated with MRS_carotid_ than the overall GrimAA measure. While we do not know the individual CpGs comprising GrimAge, we know of one CpG overlap, cg05575921, between GrimAge and MRS_carotid_, and potentially other smoking-related CpGs, which may explain the observed correlations [17].

Our results show that the epigenetic measures most strongly associated with atherosclerosis, GrimAA and MRS_carotid_, had moderate stability (ICC: 0.822 and 0.888, respectively) across repeated samples taken approximately 5 years apart. The characteristic of reliability over time is an important consideration for biomarkers that may be used for risk prediction. Further studies are needed to fully characterize the longitudinal patterns of DNA methylation, especially in response to known drivers of DNA methylation changes, such as smoking. In GENOA, smoking status was unchanged for the majority of the sample between Phases I and III, with only 27 current smokers becoming former smokers. One study that looked at the longitudinal changes of fetal DNA methylation in response to maternal smoking using serial samples at birth, age 7, and age 17 found evidence of reversible methylation changes at cg09935388 (*GFI1*) and persistent methylation changes at cg05575921 (*AHRR*) [69]. An epigenome-wide study of adult smoking reported that out of approximately 2600 CpGs that were differentially methylated between current versus never smokers, 185 CpGs showed patterns of persistent methylation changes between former versus never smokers, including cg05575921, cg09935388, and cg21161138 CpGs [85]. Most recently, Dugue et al. reported a reversibility coefficient (ratio of regression coefficients comparing former to current smokers and never to current smokers) between 69 and 75% at cg05575921, cg09935388, and cg21161138 [86]. Little is known regarding the longitudinal trends of GrimAA; however, a longitudinal trend of increased GrimAA with increasing age has been observed in one study [87].

One strength of our study is that we examined atherosclerosis at multiple vascular sites which reflect both intimal and medial vascular changes that may manifest differentially over time. CAC has been more extensively studied because it appears in a more clinically relevant vascular site [88] and is more strongly associated with coronary disease compared to carotid intima thickness [89]. Abdominal aorta atherosclerosis has been less extensively studied, yet evidence suggests that the associations of CAC and AAC with CVD are independent and additive [8, 13]. AAC starts earlier in life, is more prevalent than CAC, and may be associated with an increased risk of onset and progression of CAC and/or lower ABI [90, 91]. Given the low prevalence of CAC and previous findings that it may not carry the same pathobiological significance in African-Americans [92], incorporating extracoronary calcification may be informative and useful for risk assessment.

A limitation of our study is the attrition of participants between Phases I and III. In a previous work, we have noted that participants who were lost to follow-up had higher epigenetic age acceleration and higher CVD risk [93]. Additionally, we did not have measures of carotid plaque which was included in the multi-site atherosclerosis score that we used as a model for our score in GENOA [50], and the GENOA atherosclerosis measures were not all assessed concurrently. Additionally, seven significant CpGs from MESA were not available in GENOA because of the different arrays used. Further, we only examined *cis*-gene expression associated with DNA methylation, and thus did not capture trans-regulatory effects. Finally, gene expression was assayed in EBV transformed B-lymphoblastoid cell lines, which may not accurately reflect gene expression patterns in the white blood cells in which DNA methylation was measured. Previous findings show that significant differences exist in the DNA methylation profiles of EBV transformed lymphoblastoid cell lines and peripheral blood [94–96]. As such, gene expression profiles may also differ [95, 97], and our findings should be interpreted with caution.

## Conclusions

In conclusion, our study found evidence of associations between DNA methylation and atherosclerosis at multiple vascular sites after accounting for traditional CVD risk factors. DNA methylation changes were at CpGs with previously reported smoking-related changes. Despite being derived from CpGs associated with carotid plaque, MRS_carotid_ was associated with atherosclerosis in the coronary arteries and abdominal aorta suggesting common pathobiological mechanisms of atherosclerosis on a systemic level. One epigenetic aging measure, GrimAA, was also associated with multi-site atherosclerosis beyond traditional CVD risk factors. This is one of the very few studies to examine the DNA methylation signature of multiple atherosclerosis measures in a population-based cohort comprised solely of African-Americans. These results further our understanding of the relationship between epigenetics, biological aging, and atherosclerosis. Future work in this area, including in other racial groups, may lead to the identification of potential biomarkers of atherosclerosis, lead to better identification of those at increased risk for atherosclerosis, and further help delineate causes of cardiovascular disease disparities across populations.

## Supplementary Information

**Additional file 1: **Supplementary material, including Supplemental Tables 1–7 and Supplemental Figures 1–4.

## Data Availability

For this analysis, phenotype data are from the Database of Genotypes and Phenotypes (dbGaP): phs001401.v2.p1. Methylation data are from the Gene Expression Omnibus (GEO): GSE157131. Due to IRB restriction, mapping of the sample IDs between genotype data (dbGaP) and methylation data (GEO) cannot be provided publicly but is available upon written request to JAS and SLRK.

## Abbreviations

AAC: Abdominal aorta calcification
ABI: Ankle–brachial index
ADM: Adrenomedullin
BMI: Body mass index
CAC: Coronary artery calcification
CT: Computed tomography
CVD: Cardiovascular disease
DBP: Diastolic blood pressure
EEAA: Extrinsic epigenetic age acceleration
FDR: False discovery rate
GENOA: Genetic Epidemiology Network of Arteriopathy
ICC: Intraclass correlation coefficient
IEAA: Intrinsic epigenetic age acceleration
LDL-C: Low-density lipoprotein cholesterol
MESA: Multi-Ethnic Study of Atherosclerosis
MRS: Methylation risk score
PAI-1: Plasminogen activator inhibitor antigen type 1
PC: Principal component
SBP: Systolic blood pressure
SD: Standard deviation
TC: Total cholesterol
TIMP-1: Tissue inhibitor metalloproteinases 1
T2D: Type 2 diabetes
