# Supplementary material for: Epigenetics of single-site and multi-site atherosclerosis in African Americans from the Genetic Epidemiology Network of Arteriopathy (GENOA)
Source: Clin Epigenetics. 2022 Jan 17;14:10. doi: 10.1186/s13148-022-01229-3 (PMC8764761; doi:10.1186/s13148-022-01229-3)
Supplement: Supplementary file 1 — Additional file 1: Supplementary material, including Supplemental Tables 1–7 and Supplemental Figures 1–4. [file 13148_2022_1229_MOESM1_ESM.pdf]

Supplemental Table 1. Pearson correlations between epigenetic age acceleration measures and methylation risk scores in GENOA African Americans

	IEAA	EEAA	PhenoAA	GrimAA	MRS_{CAC}
**EEAA**	0.39*				
**PhenoAA**	0.44*	0.46*			
**GrimAA**	0.21*	0.27*	0.31*		
**MRS_{CAC}**	0.09	0.23*	0.21*	0.23*	
**$MRS_{carotid}$**	0.22*	0.28*	0.29*	0.68*	0.32*

* $P < 0.0001$
IEAA, intrinsic epigenetic age acceleration; EEAA, extrinsic epigenetic age acceleration; PhenoAA: PhenoAge acceleration; GrimAA: GrimAge acceleration

Supplemental Table 2. Association between previously-identified atherosclerosis-associated CpGs and atherosclerosis measures in GENOA African Americans (FDR < 0.1 in Model 1 for at least one atherosclerosis measure)[a]

		Multisite atherosclerosis				Coronary artery calcification score (CAC)[c]				Abdominal aorta calcification score (AAC)[c]				Ankle-brachial index (ABI)			
**CpGs**	**Previous association[b]**	**Beta**	**SE**	**P**	**FDR**	**Beta**	**SE**	**P**	**FDR**	**Beta**	**SE**	**P**	**FDR**	**Beta**	**SE**	**P**	**FDR**
cg05575921	carotid	-1.082	0.136	1.94E-14	3.48E-13	-0.576	0.121	2.75E-06	1.16E-04	-1.018	0.128	1.73E-14	1.29E-12	0.018	0.005	3.14E-04	0.003
cg09935388	carotid	-1.171	0.184	5.62E-10	5.05E-09	-0.666	0.161	4.26E-05	0.001	-0.984	0.174	3.37E-08	8.41E-07	0.025	0.006	7.93E-05	0.002
cg21161138	carotid	-2.264	0.378	4.94E-09	2.96E-08	-1.196	0.333	3.73E-04	0.005	-2.028	0.358	2.84E-08	8.41E-07	0.044	0.013	0.001	0.006
cg21566642	carotid	-1.464	0.300	1.57E-06	7.05E-06	-0.778	0.261	0.003	0.026	-1.511	0.281	1.29E-07	2.42E-06	0.013	0.010	0.193	
cg01940273	carotid	-1.416	0.342	4.37E-05	1.30E-04	-0.638	0.296	0.032	0.081	-1.477	0.321	5.67E-06	8.51E-05	0.012	0.012	0.305	
cg08958747	carotid	-1.954	0.474	4.62E-05	1.30E-04	-1.071	0.409	0.009	0.039	-0.977	0.454	0.032		0.044	0.016	0.007	0.030
cg14753356	carotid	-1.771	0.432	5.07E-05	1.30E-04	-1.305	0.372	0.000	0.005	-1.380	0.408	0.001	0.006	0.024	0.015	0.108	
cg24859433	carotid	-1.115	0.295	1.82E-04	4.09E-04	-0.698	0.256	0.007	0.032	-1.012	0.278	0.000	0.003	0.018	0.010	0.072	
cg19572487	carotid	-1.305	0.351	2.34E-04	4.67E-04	-0.638	0.305	0.037	0.087	-1.327	0.329	0.000	0.001	0.024	0.012	0.044	0.067
cg03636183	carotid	-0.913	0.249	2.86E-04	0.001	-0.518	0.212	0.015	0.053	-0.851	0.235	0.000	0.003	0.011	0.009	0.193	
cg15342087	carotid	-1.111	0.325	0.001	0.001	-0.783	0.281	0.006	0.029	-0.939	0.307	0.002	0.016	0.022	0.011	0.041	0.067
cg25953130	carotid	-0.956	0.279	0.001	0.001	-0.465	0.239	0.052		-0.677	0.263	0.011	0.056	0.019	0.009	0.042	0.067
cg18168448	carotid	-1.005	0.310	0.001	0.002	-0.676	0.265	0.011	0.043	-0.544	0.294	0.065		0.014	0.011	0.182	
cg04761231	carotid	-1.777	0.600	0.003	0.004	-0.533	0.514	0.300		-1.244	0.568	0.029		0.030	0.020	0.138	
cg18446336	carotid	-0.711	0.239	0.003	0.004	-0.437	0.204	0.033	0.081	-0.810	0.224	0.000	0.003	0.002	0.008	0.827	
cg19979108	carotid	-1.153	0.411	0.005	0.006	-0.628	0.352	0.075		-0.645	0.389	0.098		0.032	0.014	0.022	0.054
cg13200854	carotid	-1.336	0.479	0.006	0.006	-1.195	0.413	0.004	0.028	-0.519	0.454	0.254		0.018	0.016	0.262	
cg09646173	carotid	-1.116	0.409	0.007	0.007	-0.542	0.354	0.127		-0.347	0.388	0.372		0.023	0.014	0.096	
cg03295554	carotid	-0.795	0.302	0.009	0.008	-0.370	0.258	0.153		-0.776	0.284	0.007	0.038	0.007	0.010	0.471	
cg21271420	carotid	-1.434	0.547	0.009	0.008	-1.013	0.471	0.032	0.081	-0.672	0.518	0.195		0.030	0.018	0.110	
cg26712743	carotid	-1.439	0.556	0.010	0.009	-1.078	0.473	0.023	0.076	-0.695	0.523	0.185		0.010	0.019	0.589	
cg24736734	carotid	-1.733	0.685	0.012	0.010	-1.196	0.589	0.043	0.087	-1.033	0.647	0.111		0.041	0.023	0.078	
cg05246522	CAC	-0.881	0.362	0.015	0.012	-0.497	0.311	0.111		-0.410	0.343	0.232		0.005	0.012	0.657	
cg03738331	carotid	1.009	0.437	0.021	0.016	0.247	0.377	0.512		1.044	0.410	0.011	0.056	-0.031	0.015	0.037	0.067
cg15501219	carotid	-1.939	0.857	0.024	0.017	-1.139	0.737	0.123		-0.231	0.812	0.776		0.049	0.029	0.089	
cg00294684	carotid	-1.074	0.486	0.028	0.019	-0.613	0.419	0.144		-0.294	0.460	0.523		0.023	0.016	0.153	
cg22773522	carotid	-0.752	0.370	0.042	0.028	-0.398	0.317	0.210		-0.532	0.349	0.128		0.021	0.012	0.093	
cg10512376	carotid	-0.847	0.427	0.048	0.031	-0.071	0.366	0.845		-0.334	0.403	0.407		0.020	0.014	0.156	
cg03072035	carotid	-0.817	0.429	0.058	0.035	-0.109	0.372	0.770		-0.412	0.402	0.306		0.012	0.014	0.411	
cg05119988	CAC	-0.744	0.390	0.057	0.035	-0.381	0.328	0.247		-0.603	0.368	0.103		0.000	0.013	0.973	
cg10508317	carotid	-0.616	0.336	0.068	0.039	-0.363	0.285	0.204		-0.072	0.318	0.822		0.012	0.011	0.294	
cg12547807	carotid	-1.002	0.552	0.070	0.039	-0.402	0.471	0.395		-0.706	0.520	0.176		0.051	0.018	0.006	0.030
cg00834988	carotid	-0.842	0.485	0.083	0.045	-0.584	0.414	0.159		-0.276	0.458	0.547		0.027	0.016	0.100	
cg03355101	carotid	-0.429	0.265	0.107	0.053	-0.286	0.228	0.211		-0.316	0.250	0.206		0.006	0.009	0.488	
cg14026106	CAC	-0.591	0.360	0.102	0.053	-0.497	0.309	0.108		-0.179	0.339	0.597		0.003	0.012	0.784	
cg16661609	carotid	-0.891	0.551	0.106	0.053	-0.200	0.473	0.672		-1.107	0.517	0.033		0.005	0.019	0.769	
cg03928367	carotid	-1.129	0.733	0.125	0.060	-1.276	0.628	0.043	0.087	-0.981	0.686	0.154		0.012	0.024	0.614	
cg07033253	carotid/CAC	-0.603	0.394	0.127	0.060	-0.515	0.339	0.130		-0.806	0.369	0.030		-0.010	0.013	0.441	
cg19266329	carotid	-0.542	0.371	0.145	0.067	-0.423	0.318	0.184		-0.607	0.349	0.083		0.005	0.013	0.682	
cg12912426	carotid	-0.648	0.468	0.167	0.075	0.096	0.404	0.813		-0.362	0.441	0.412		0.013	0.016	0.411	
cg18561976	carotid	-0.267	0.202	0.187	0.082	-0.179	0.171	0.297		-0.342	0.190	0.073		0.003	0.007	0.670	
cg15344028	carotid	-0.361	0.278	0.196	0.084	-0.295	0.234	0.209		-0.759	0.260	0.004	0.023	-0.006	0.009	0.498	
cg01110839	carotid	0.569	0.495	0.251		0.938	0.423	0.027	0.081	0.370	0.466	0.427		-0.005	0.017	0.775	
cg24420089	CAC	0.417	0.532	0.434		0.911	0.449	0.043	0.087	0.376	0.501	0.454		0.015	0.018	0.410	
cg16107001	carotid	0.477	0.659	0.470		-0.328	0.570	0.565		0.342	0.619	0.581		-0.057	0.022	0.009	0.030
cg12798040	CAC	-0.270	0.419	0.519		0.137	0.355	0.700		-0.052	0.394	0.894		0.029	0.014	0.043	0.067
cg20507228	carotid	0.171	0.308	0.580		0.729	0.262	0.006	0.029	0.592	0.288	0.041		0.026	0.010	0.012	0.035
cg13913475	CAC	0.004	0.074	0.958		-0.034	0.063	0.596		-0.044	0.070	0.529		-0.006	0.002	0.023	0.054

Model 1 is adjusted for age, sex, time between measures, and first 4 genetic principal components
Beta is the change in the atherosclerosis measure associated with a 1 unit increase in the CpG methylation.
FDR ≥ 0.10 are left blank
[a] Only associations with FDR < 0.1 in Model 1 for any of the four measures of atherosclerosis are shown.
[b] Associations from: Liu Y, Reynolds LM, Ding J, Hou L, Lohman K, Young T, et al. Blood monocyte transcriptome and epigenome analyses reveal loci associated with human atherosclerosis. Nat Commun. 2017;8(1):393.
[c] Coronary artery and abdominal aorta calcification scores were transformed as ln[(CAC+1)] and ln[(AAC+1)]

Supplemental Table 3. Association between previously-identified atherosclerosis-associated CpGs and atherosclerosis measures in GENOA African Americans (FDR < 0.1 in Model 3 for at least one atherosclerosis measure)[a]

		Multisite atherosclerosis				Coronary artery calcification score (CAC)[c]				Abdominal aorta calcification score (AAC)[c]				Ankle-brachial index (ABI)			
CpGs	Previous association[b]	Beta	SE	P	FDR	Beta	SE	P	FDR	Beta	SE	P	FDR	Beta	SE	P	FDR
cg05575921	carotid	-0.968	0.200	1.97E-06	1.47E-04	-0.496	0.174	0.005	0.151	-0.900	0.187	2.15E-06	1.47E-04	0.019	0.007	0.010	0.188
cg09935388	carotid	-0.700	0.206	0.001	0.027	-0.343	0.176	0.052	0.292	-0.515	0.192	0.008	0.134	0.023	0.007	0.002	0.099
cg21161138	carotid	-1.314	0.443	0.003	0.063	-0.567	0.380	0.136	0.525	-1.209	0.413	0.004	0.124	0.036	0.016	0.023	0.228
cg18168448	carotid	-0.868	0.294	0.003	0.063	-0.502	0.250	0.045	0.292	-0.493	0.275	0.073	0.418	0.012	0.011	0.248	0.657

Model 3 is adjusted for age, sex, time between measures, first 4 genetic principal components, smoking, T2D status, hypertension status, BMI, and statin-adjusted total cholesterol levels.

Beta is the change in the atherosclerosis measure associated with a 1 unit increase in the CpG methylation.

[a] Only associations with FDR < 0.1 in Model 3 for any of the four measures of atherosclerosis are shown.

[b] Associations from: Liu Y, Reynolds LM, Ding J, Hou L, Lohman K, Young T, et al. Blood monocyte transcriptome and epigenome analyses reveal loci associated with human atherosclerosis. Nat Commun. 2017;8(1):393.

[c] Coronary artery and abdominal aorta calcification scores were transformed as ln[(CAC+1)] and ln[(AAC+1)]

Supplemental Table 4. DNA methylation associated with cis-gene expression and atherosclerosis in GENOA African Americans (N=349)

CpG methylation characteristics				Cis- mRNA gene expression ~ CpG methylation[a]					Multisite atherosclerosis ~ mRNA gene expression			CAC[a] ~ mRNA gene expression			AAC[a] ~ mRNA gene expression			ABI ~ mRNA gene expression		
**CpG**	**Chromosome**	**Location**	**Atherosclerosis measure association**	**Gene**	**correlation**	**Beta**	**P**	**FDR**	**Beta**	**SE**	**P**	**Beta**	**SE**	**P**	**Beta**	**SE**	**P**	**Beta**	**SE**	**P**
cg18168448	1	*LRRC52* (1st exon)	MA, CAC	*ALDH9A1*	-0.14	-0.06	0.005	0.064	1.62	0.84	0.054	**2.00**	**0.71**	**0.005**	0.78	0.79	0.323	0.02	0.29	0.518
cg18168448	1	*LRRC52* (1st exon)	MA, CAC	ENSG00000236364	0.15	0.06	0.003	0.064	-1.39	0.87	0.110	-0.54	0.75	0.467	-0.94	0.82	0.251	0.03	0.03	0.318
cg03636183	19	*F2RL3* (Body)	MA, CAC, AAC	*F2RL3*	-0.18	-0.05	0.001	0.062	-1.41	0.90	0.119	**-1.82**	**0.76**	**0.017**	-0.64	0.85	0.852	-0.003	0.03	0.918

MA: multisite atherosclerosis; CAC: coronary artery calcification; AAC: abdominal aorta calcification; ABI: ankle-brachial index
Table includes CpGs with significant (FDR < 0.1) associations between peripheral blood CpG methylation and cis (± 1MB) mRNA gene expression profiles in lymphoblastoid cell lines, and atherosclerosis measures in Models adjusted for age, sex, time between measures, and the first 4 genetic PCs
[a] Coronary artery and abdominal aorta calcification scores were transformed as ln[(CAC+1)] and ln[(AAC+1)]

Supplemental Table 5. Association between epigenetic age acceleration measures and single and multisite atherosclerosis after adjusting for white blood cell counts in GENOA African Americans

Outcome	Epigenetic age acceleration	Model 1			Model 2			Model 3		
		Beta	SE	*P*	Beta	SE	*P*	Beta	SE	*P*
Coronary artery calcification score (CAC)[a]	PhenoAA	0.041	0.019	0.030	0.024	0.019	0.202	0.010	0.018	0.595
	GrimAA	**0.149**	**0.029**	**2.85×10^{-7}**	**0.108**	**0.036**	**0.003**	0.073	0.035	0.036
Abdominal aorta calcification score (AAC)[a]	PhenoAA	**0.052**	**0.020**	**0.012**	0.029	0.020	0.149	0.020	0.019	0.300
	GrimAA	**0.194**	**0.031**	**1.07×10^{-9}**	**0.106**	**0.038**	**0.005**	**0.087**	**0.038**	**0.023**
Ankle-brachial index (ABI)	PhenoAA	-0.001	0.001	0.069	-0.001	0.001	0.135	-0.001	0.001	0.135
	GrimAA	**-0.003**	**0.001**	**0.002**	-0.003	0.001	0.016	-0.004	0.001	0.016
Multisite atherosclerosis score	PhenoAA	**0.074**	**0.022**	**0.001**	0.050	0.021	0.019	0.040	0.021	0.054
	GrimAA	**0.231**	**0.033**	**7.25×10^{-11}**	**0.157**	**0.040**	**1.08×10^{-4}**	**0.137**	**0.040**	**7.87×10^{-4}**

PhenoAA: PhenoAge acceleration; GrimAA: GrimAge acceleration
Model 1 is adjusted for age, sex, time between measures, and 5 white blood cell counts
Model 2 is adjusted for Model 1 covariates and smoking status
Model 3 is adjusted for Model 2 covariates, hypertension status, diabetes status, body mass index, and total cholesterol levels adjusted for lipid lowering medications
Beta is the change in the atherosclerosis measure associated with a 1-year increase in the epigenetic age acceleration measure.
Association with $P < 0.05$ are shown in bold font
[a] Coronary artery and abdominal aorta calcification scores were transformed as ln[(CAC+1)] and ln[(AAC+1)]

Supplemental Table 6. Association between components of GrimAge and single and multisite atherosclerosis in GENOA African Americans

	DNAm Adrenomedullin (ADM)			DNAm Beta-2-microglobulim (B2M)			DNAm Cystatin C			DNAm Growth Differentiation Factor 15 (GDF15)			DNAm Leptin			DNAm Smoking pack-years			DNAm Plasminogen activator inhibitor antigen type 1 (PAI1)			DNAm Tissue inhibitor metalloproteinases 1 (TIMP1)		
**Outcome**	**Beta**	**SE**	***P***	**Beta**	**SE**	***P***	**Beta**	**SE**	***P***	**Beta**	**SE**	***P***	**Beta**	**SE**	***P***	**Beta**	**SE**	***P***	**Beta**	**SE**	***P***	**Beta**	**SE**	***P***
Coronary artery calcification score (CAC)[a]	0.333	0.182	0.068	**0.372**	**0.167**	**0.026**	**0.558**	**0.230**	**0.016**	**0.354**	**0.158**	**0.026**	0.424	0.238	0.076	**0.569**	**0.132**	**2.21E-05**	**0.457**	**0.127**	**3.48E-04**	0.413	0.216	0.058
Abdominal aorta calcification score (AAC)[a]	0.037	0.200	0.853	**0.582**	**0.184**	**0.002**	0.393	0.256	0.126	**0.512**	**0.174**	**0.003**	0.175	0.260	0.502	**1.050**	**0.139**	**3.19E-13**	**0.282**	**0.141**	**0.046**	-0.013	0.241	0.958
Ankle brachial index (ABI)	-0.012	0.007	0.104	-0.013	0.007	0.052	**-0.035**	**0.009**	**1.12E-04**	-0.003	0.006	0.579	4.68E-04	0.009	0.959	**-0.012**	**0.005**	**0.024**	0.001	0.005	0.892	-0.009	0.009	0.288
Multisite atherosclerosis score	**0.472**	**0.211**	**0.026**	**0.557**	**0.195**	**0.005**	**0.859**	**0.269**	**0.002**	**0.431**	**0.185**	**0.021**	0.281	0.276	0.310	**1.021**	**0.149**	**3.23E-11**	**0.447**	**0.148**	**0.003**	0.345	0.256	0.178

Model is adjusted for age, sex, time between measures, and white blood cell counts
Beta is change in the atherosclerosis measure associated with a 1 standard deviation increase in the scaled and centered GrimAge component.
Association with $P < 0.05$ are shown in bold font
[a] Coronary artery and abdominal aorta calcification scores were transformed as ln[(CAC+1)] and ln[(AAC+1)]

Supplemental Table 7. Association between methylation risk scores at Phase II and atherosclerosis measures in GENOA African Americans (N = 129)

Outcome	Epigenetic measure	Beta	SE	*P*
Coronary artery calcification score (CAC) [a]	MRS_{CAC}	0.008	0.261	0.976
	$MRS_{carotid}$	0.178	0.336	0.598
Abdominal aorta calcification score (AAC) [a]	MRS_{CAC}	0.232	0.284	0.416
	$MRS_{carotid}$	**1.08**	**0.357**	**0.003**
Ankle Brachial Index (ABI)	MRS_{CAC}	0.005	0.009	0.561
	$MRS_{carotid}$	-0.002	0.012	0.834
Multisite atherosclerosis score	MRS_{CAC}	0.029	0.288	0.920
	$MRS_{carotid}$	0.811	0.368	0.030

Model is adjusted for age, sex, time between measures (when applicable), first 4 genetic principal components, smoking status, hypertension status, diabetes status, body mass index, and total cholesterol levels adjusted for statin use.
Beta is the change in the atherosclerosis measure associated with a 1 unit increase in the MRS.
P values significant after Bonferroni correction ($P < 0.025$) are shown in bold font
[a] Coronary artery and abdominal aorta calcification scores were transformed as ln[(CAC+1)] and ln[(AAC+1)]

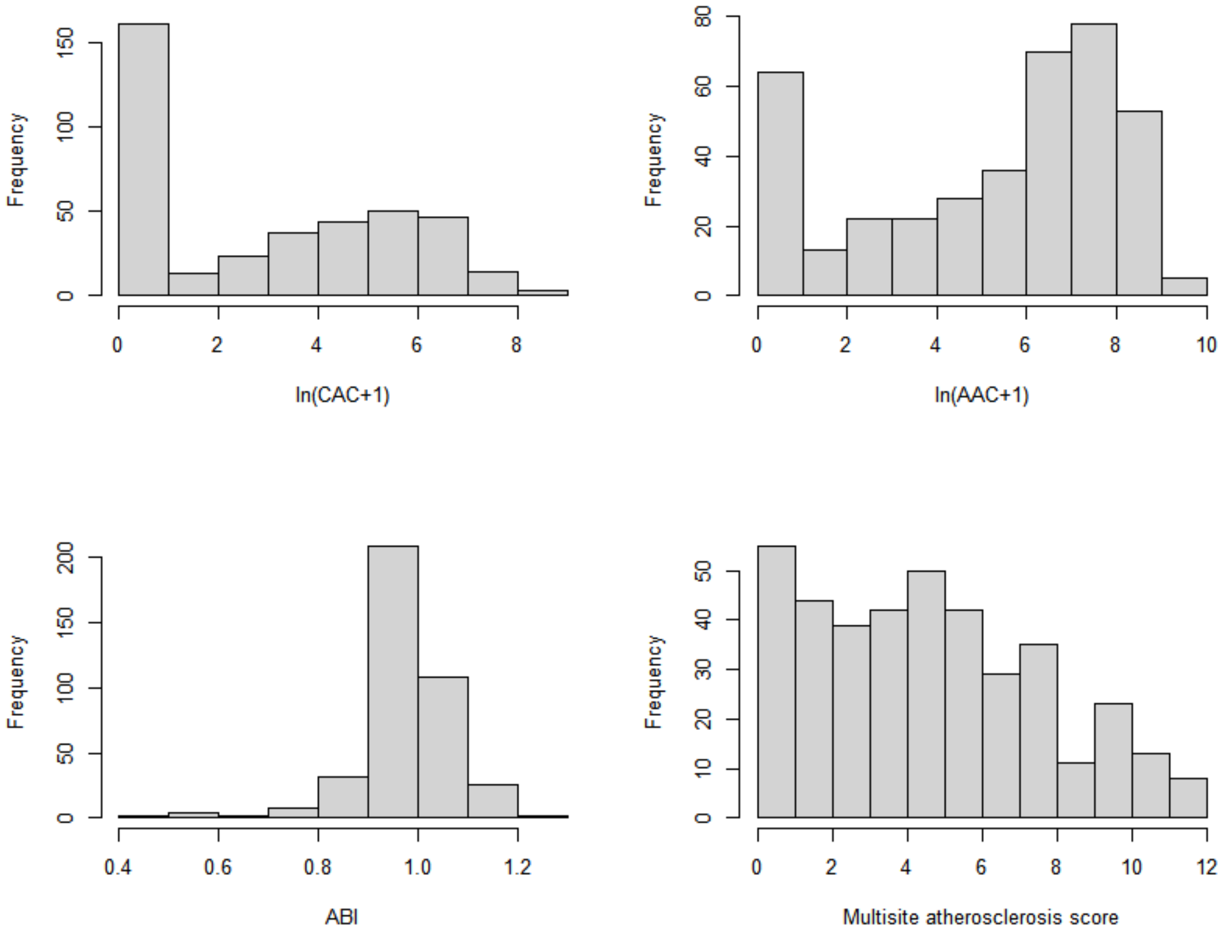

Supplemental Figure 1. Distribution of single- and multi-site atherosclerosis measures in GENOA African Americans

CAC, coronary artery calcification; AAC, abdominal aorta calcification; ABI, ankle-brachial index

Supplemental Figure 2. Distribution of MRS_{CAC} and $MRS_{carotid}$ in GENOA African Americans

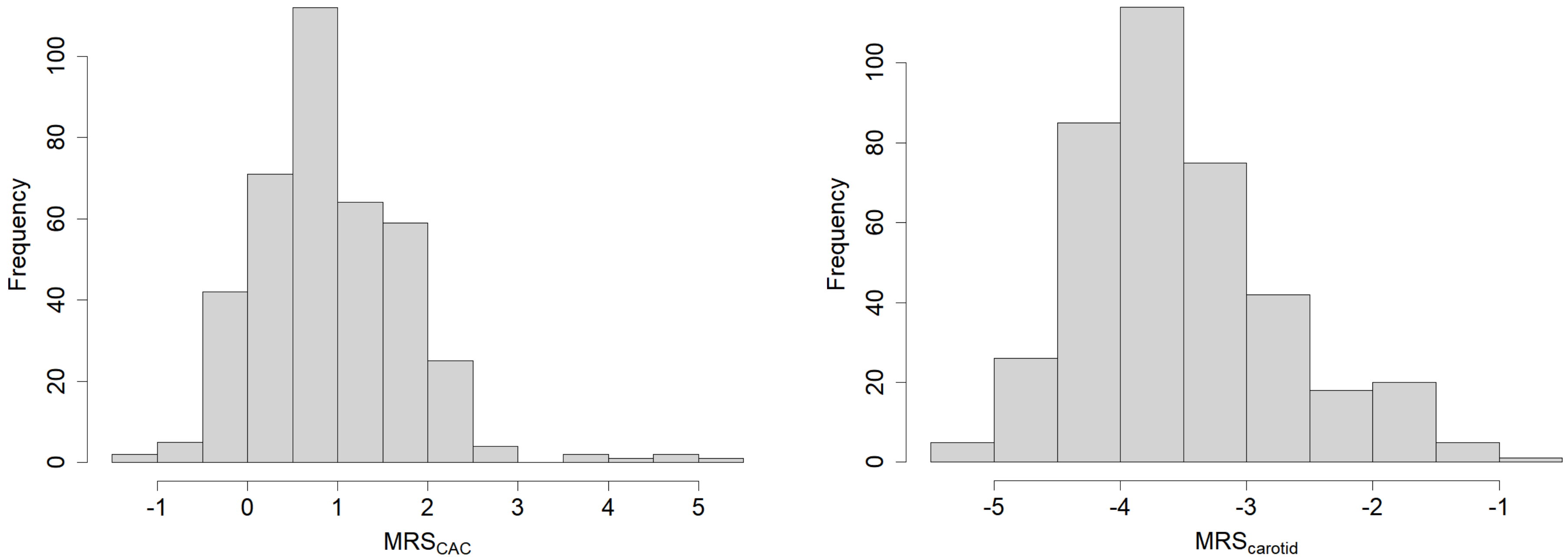

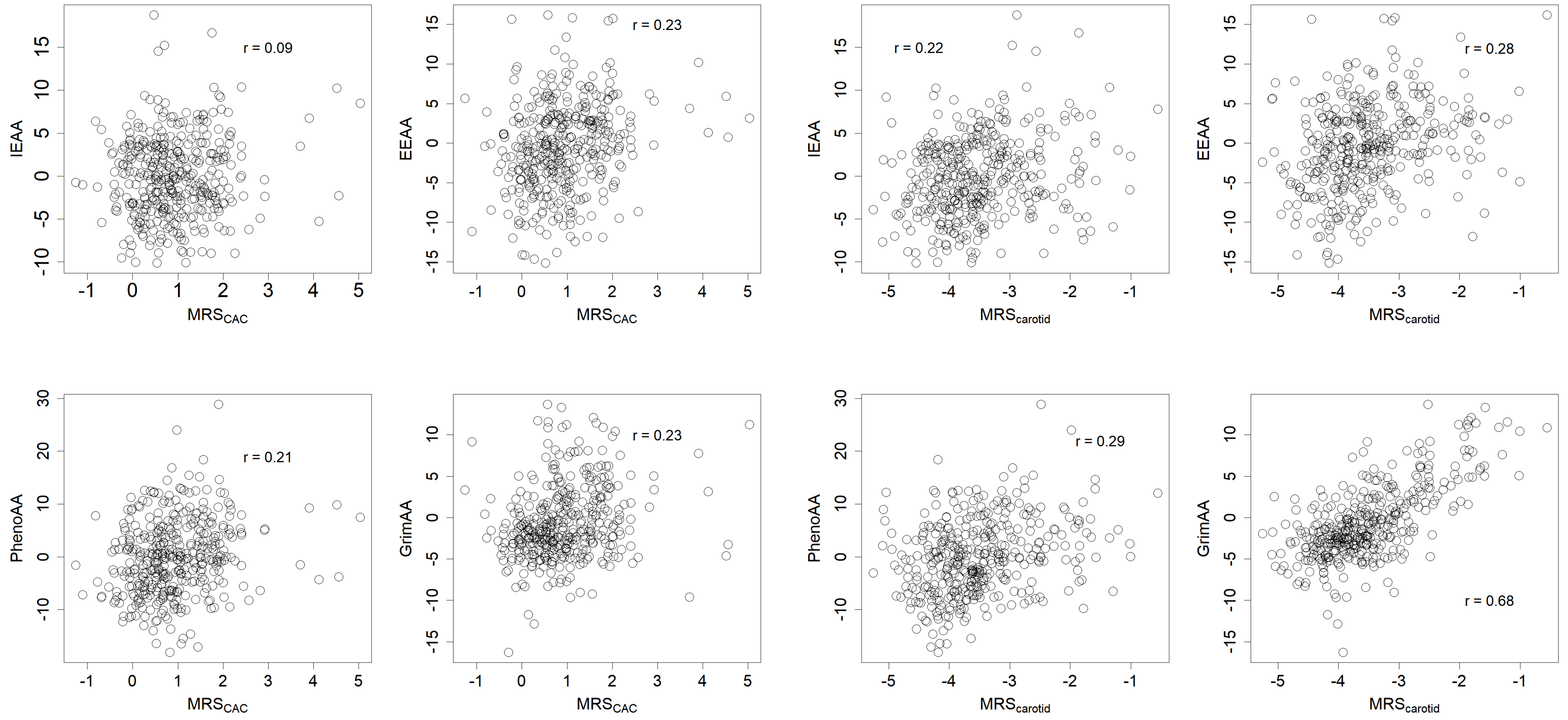

Supplemental Figure 3. Scatterplots and Pearson correlation coefficients for methylation risk scores (MRS_{CAC} and $MRS_{carotid}$) and epigenetic age acceleration (IEAA, EEAA, PhenoAA, and GrimAA) in GENOA African Americans.

IEAA, intrinsic epigenetic age acceleration; EEAA, extrinsic epigenetic age acceleration; PhenoAA: PhenoAge acceleration; GrimAA: GrimAge acceleration

A

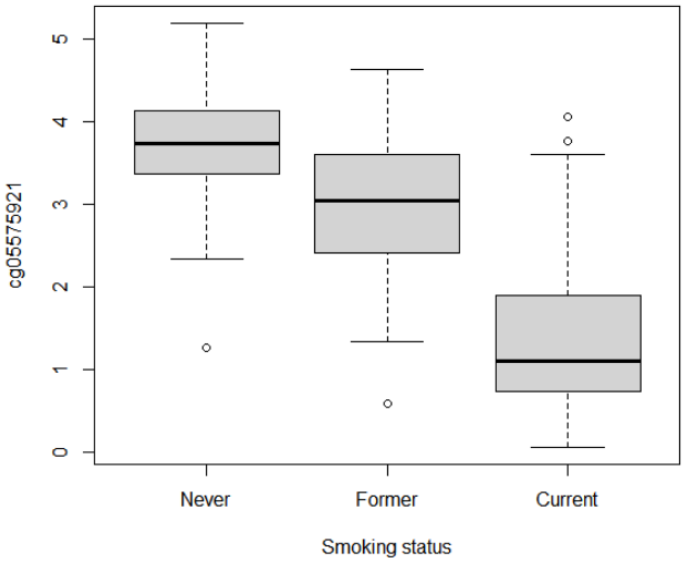

B

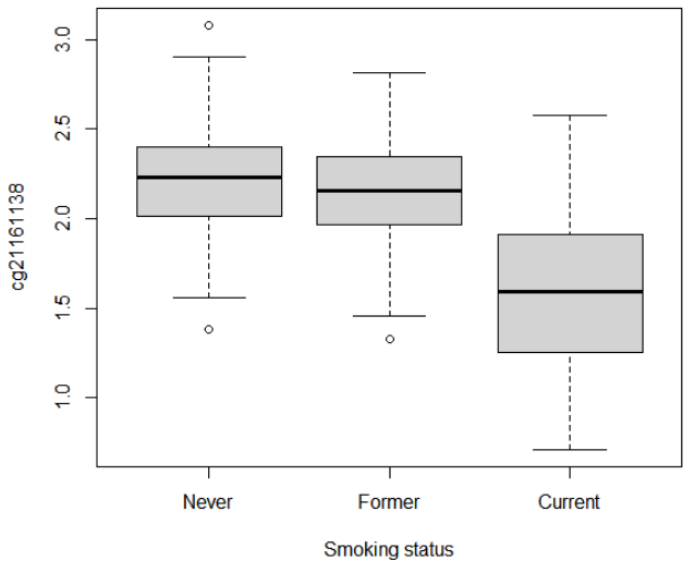

C

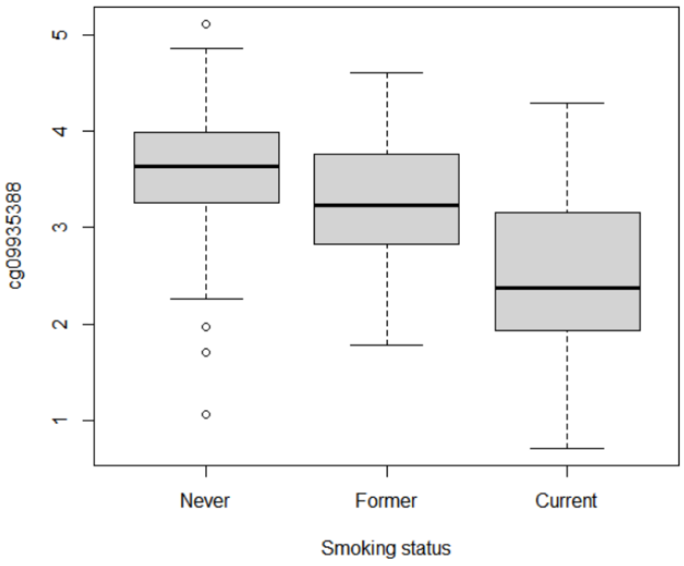

Supplemental Figure 4. Methylation at the cg05575921 (A), cg21161138 (B) and cg09935388 (C) by smoking status at Phase I in GENOA African Americans
